# Uncovering convolutional neural network decisions for diagnosing multiple sclerosis on conventional MRI using layer-wise relevance propagation

**DOI:** 10.1016/j.nicl.2019.102003

**Published:** 2019-09-06

**Authors:** Fabian Eitel, Emily Soehler, Judith Bellmann-Strobl, Alexander U. Brandt, Klemens Ruprecht, René M. Giess, Joseph Kuchling, Susanna Asseyer, Martin Weygandt, John-Dylan Haynes, Michael Scheel, Friedemann Paul, Kerstin Ritter

**Affiliations:** a Charité – Universitätsmedizin Berlin, Freie Universität Berlin, Humboldt-Universität zu Berlin, Berlin Institute of Health (BIH), Department of Psychiatry and Psychotherapy, 10117 Berlin, Germany; b Charité – Universitätsmedizin Berlin, Freie Universität Berlin, Humboldt-Universität zu Berlin, Berlin Institute of Health (BIH), Berlin Center for Advanced Neuroimaging, Bernstein Center for Computational Neuroscience, 10117 Berlin, Germany; c Charité – Universitätsmedizin Berlin, Freie Universität Berlin, Humboldt-Universität zu Berlin, Berlin Institute of Health (BIH), Department of Neurology, 10117 Berlin, Germany; d Charité – Universitätsmedizin Berlin, Freie Universität Berlin, Humboldt-Universität zu Berlin, Berlin Institute of Health (BIH), NeuroCure Clinical Research Center, 10117 Berlin, Germany; e Charité – Universitätsmedizin Berlin, Freie Universität Berlin, Humboldt-Universitt zu Berlin, Berlin Institute of Health (BIH), Experimental and Clinical Research Center, Max Delbrück Center for Molecular Medicine, 10117 Berlin, Germany; f Charité – Universitätsmedizin Berlin, Freie Universität Berlin, Humboldt-Universitt zu Berlin, Berlin Institute of Health (BIH), Department of Neuroradiology, 10117 Berlin, Germany; g Einstein Center for Digital Future Berlin, Germany; h Department of Neurology, University of California, Irvine, CA, USA

**Keywords:** Convolutional neural networks deep learning multiple sclerosis MRI, Layer-wise relevance propagation, Visualization transfer learning

## Abstract

Machine learning-based imaging diagnostics has recently reached or even surpassed the level of clinical experts in several clinical domains. However, classification decisions of a trained machine learning system are typically non-transparent, a major hindrance for clinical integration, error tracking or knowledge discovery. In this study, we present a transparent deep learning framework relying on 3D convolutional neural networks (CNNs) and layer-wise relevance propagation (LRP) for diagnosing multiple sclerosis (MS), the most widespread autoimmune neuroinflammatory disease. MS is commonly diagnosed utilizing a combination of clinical presentation and conventional magnetic resonance imaging (MRI), specifically the occurrence and presentation of white matter lesions in T2-weighted images. We hypothesized that using LRP in a naive predictive model would enable us to uncover relevant image features that a trained CNN uses for decision-making. Since imaging markers in MS are well-established this would enable us to validate the respective CNN model. First, we pre-trained a CNN on MRI data from the Alzheimer's Disease Neuroimaging Initiative (*n* = 921), afterwards specializing the CNN to discriminate between MS patients (*n* = 76) and healthy controls (*n* = 71). Using LRP, we then produced a heatmap for each subject in the holdout set depicting the voxel-wise relevance for a particular classification decision. The resulting CNN model resulted in a balanced accuracy of 87.04% and an area under the curve of 96.08% in a receiver operating characteristic curve. The subsequent LRP visualization revealed that the CNN model focuses indeed on individual lesions, but also incorporates additional information such as lesion location, non-lesional white matter or gray matter areas such as the thalamus, which are established conventional and advanced MRI markers in MS. We conclude that LRP and the proposed framework have the capability to make diagnostic decisions of CNN models transparent, which could serve to justify classification decisions for clinical review, verify diagnosis-relevant features and potentially gather new disease knowledge.

## Highlights

• LRP helps in explaining individual CNN decisions for diagnosing multiple sclerosis (MS) based on conventional MRI data
• CNNs learn to identify hyperintense lesions as an important biomarker of MS
• CNNs learn to identify relevant areas beyond lesions
• Transfer learning improves learning across diseases and MRI sequences
• Transparent CNNs show potential in validating models, verifying diagnosis-relevant features and gathering disease knowledge

## 1 Introduction

Multiple Sclerosis (MS) is the most widespread autoimmune neuroinflammatory disease in young adults with 2.2 million cases reported worldwide (Mitchell et al., 2019). The disease is mainly characterized by inflammation, demyelination and neurodegeneration in the central nervous system and often leads to substantial disability in patients (Reich et al., 2018). The current quasi-standard for diagnosing MS, the McDonald criteria, relies on clinical presentation and the presence of lesions visible in conventional T2-weighted brain magnetic resonance imaging (MRI) data (Thompson et al., 2018). Most common in clinical practice are fluid-suppressed T2-weighted image sequences (e.g. fluid-attenuated inversion recovery sequence [FLAIR]), which are sensitive towards MS-relevant white matter lesions, but also relatively unspecific with respect to underlying disease processes (Geraldes et al., 2018). Several other imaging markers have been described including global brain atrophy, thalamic atrophy, cortical lesions, altered structural and functional connectivity or central vein signs (Lowe et al., 2002; Azevedo et al., 2018; Absinta et al., 2016; Filippi et al., 2016; Sinnecker et al., 2019; Backner et al., 2018; Pawlitzki et al., 2017; Solomon et al., 2017), of which some are captured in conventional MRI and others require advanced MRI techniques such as diffusion weighted imaging or functional MRI.

In the last decade, a lot of research effort has been put on the automatic (i.e. data-driven) detection of neurological diseases based on neuroimaging data including MRI (Orrù et al., 2012; Woo et al., 2017). Early approaches combined parameter-based machine learning algorithms, such as support vector machines, with carefully extracted features known or hypothesized to be relevant in the respective disease. In MS research, features ranging from T2 lesion characteristics to atrophy to local intensity patterns or multi-scale information extracted from MRI data have been used in combination with standard machine learning analyses to either diagnose MS or predict disease progression (Eshaghi et al., 2018; Nichols et al., 2012; Weygandt et al., 2011; Hackmack et al., 2012a; Hackmack et al., 2012b; Weygandt et al., 2015; Wottschel et al., 2015). While choosing features based on expert criteria reflects the current state of knowledge, it does not allow for finding new and potentially unexpected hidden data properties, which might also help in characterizing a certain disease. Deep learning techniques fill a gap here and allow for utilizing hierarchical information directly from raw or minimally processed data (Lecun et al., 2015). By being specifically tailored to image data, in particular convolutional neural networks (CNNs) have led to major breakthroughs in medical imaging (Litjens et al., 2017; Rajpurkar et al., 2017a; Rajpurkar et al., 2017b; De Fauw et al., 2018). In neuroimaging, most CNN analyses so far focused on Alzheimer's disease (Vieira et al., 2017), but there are also some recent studies in MS. Given the importance of lesions in diagnosing MS and monitoring disease progression, most efforts have been put on the task of lesion segmentation (Valverde et al., 2017; Li et al., 2016; Khastavaneh and Ebrahimpour-Komleh, 2017). Others used CNNs to diagnose MS based on 2-dimensional MRI slices (Wang et al., 2018) or to predict short-term disease activity based on binary lesion masks (Yoo et al., 2016).

Despite their potential, deep learning methods are criticized for being non-transparent (such as a ‘black box’) due to the difficulty to retrace the classification decision in light of huge parameter spaces and highly non-linear interactions (Castelvecchi, 2016). This is especially problematic in medical applications since understanding and explaining neural network decisions is required for clinical integration, error tracking or knowledge discovery. Explaining neural network decisions is an open research area in computer science and a number of suggestions have been made in recent years. Different directions for explanations include visualizing features (Zeiler and Fergus, 2014), generating images that maximally activate a certain neuron (Olah et al., 2017) and creating heatmaps based on the input images indicating the relevance of each voxel for the final classification decision (Simonyan and Zisserman, 2014; Bach et al., 2015; Springenberg et al., 2015). Heatmaps are in particular valuable in the medical context, since they allow for an easy and intuitive investigation of what the respective classifier found to be important directly in the input data. Besides understanding diagnostic decisions for individual patients, heatmaps might be useful in validating CNN models. Recently, we have shown the potential of transparent CNN applications for knowledge discovery in Alzheimer's disease (Rieke et al., 2018; Böhle et al., 2019).

**Fig. 1:**
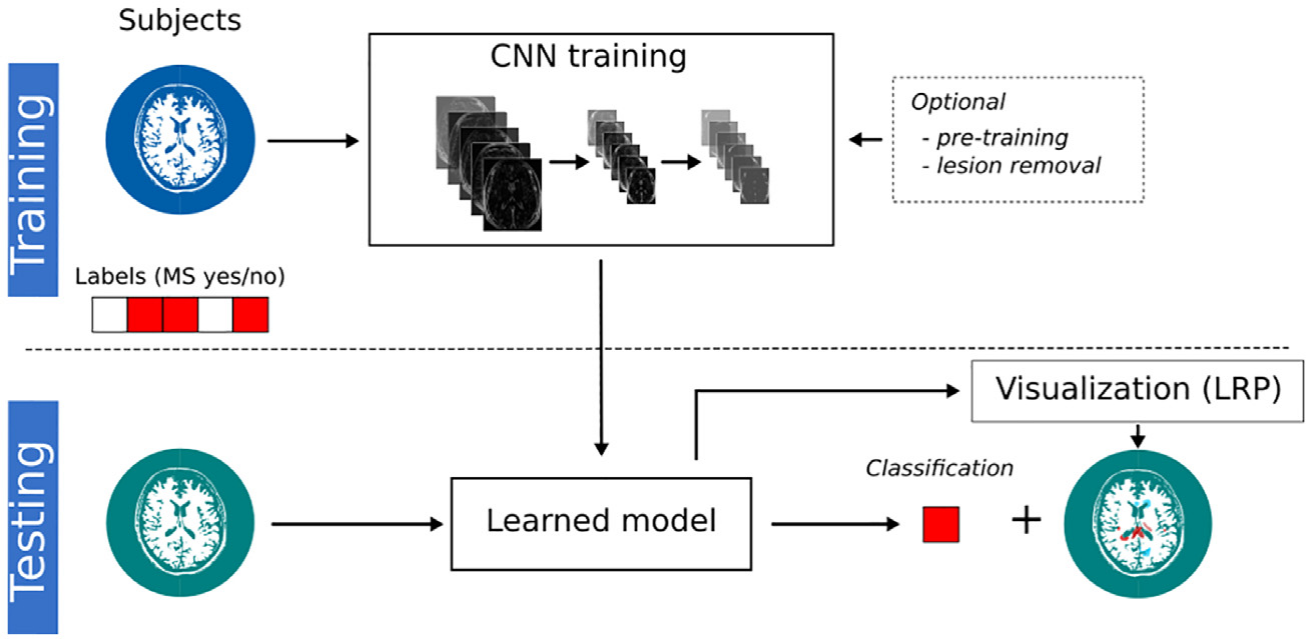
Illustration of the transparent CNN framework. In the training phase, the CNN model learns a non-linear relationship between the MRI data and the binary diagnostic labels (MS yes/no). Optionally, the CNN models are pre-trained on a substitute data set or lesions are filled in the MRI data. The learned CNN model is then tested on new subjects to predict the diagnostic label. By supplementing this label with a LRP heatmap, which indicates the relevance of each voxel for the respective label, this framework allows us to understand (at least to some extent) the classification decision in individual subjects. Additionally, the validity of the CNN models can be assessed by matching highlighted brain areas with domain knowledge.

The objective of the current study was to investigate whether a transparency approach can uncover decision processes in MRI-based diagnosis of MS, a disease with well-defined imaging markers, thereby supporting future clinical implementation and verification of machine learning-based diagnosis systems. We present a transparent CNN framework (see Fig. 1) for the MRI-based diagnosis of MS relying on layer-wise relevance propagation (LRP, (Bach et al., 2015; Samek et al., 2017a)) – a heatmap method that has been shown to outperform previous approaches in terms of explainability and disease-specific evidence (Böhle et al., 2019; Samek et al., 2017a). Since the data set was rather small (*n* = 147), we investigated the effect of pre-training the CNN on data from the Alzheimer's Disease Neuroimaging Initiative (ADNI, *n* = 921). Using LRP, individual heatmaps were generated for each subject and analyzed with respect to well-established imaging features in MS (e.g. white matter lesions or thalamic atrophy). By showing that LRP in combination with a naive CNN model (i.e. a model independent of MS-specific knowledge) indeed helps in uncovering relevant imaging features, we conclude that this framework is not only useful in justifying individual diagnostic decisions but also to validate CNN models (especially in light of small sample sizes).

## 2 Materials and methods

### 2.1 Subjects

In the present study, we retrospectively analyzed data collected by FP from Charité – Universitätsmedizin Berlin as part of the VIMS study: Follow-up examination of visual parameters for the creation of a database (neuro-ophthalmologic register) in patients with MS versus healthy subjects.^2^ We enrolled 76 patients with relapsing-remitting MS according to the McDonald criteria 2010 (Polman et al., 2011) and 71 healthy controls. Patients were excluded if they were outside the age range of 18–69 or did not have an MRI scan. All patients were examined under supervision of a board-certified neurologist at the NeuroCure Clinical Research Center (Charité – Universitätsmedizin Berlin) between January 2011 and July 2015. All participants provided written informed consent prior to their inclusion in the study. The study was approved by the local ethics committee and was performed in accordance with the 1964 Declaration of Helsinki in its currently applicable version. Part of this data has been used in previous studies (e.g. (Kuchling et al., 2018)). Demographical details of subjects can be found in Table 1. There is a significant group difference in age (*p* < 0.05, obtained via a *t*-test), but not in sex (chi-squared test).

**Table 1:** Demographics of MS patients and healthy controls. Disease duration is measured in months and lesion volume in ml. EDSS, expanded disability status scale; std., standard deviation.

	MS patients	Healthy controls
Subjects [n]	76	71
Female/Male, in %	55% / 45%	65% / 35%
Age (in years), mean ± std	43.32 (± 11.99)	38.23 (± 13.10)
Disease duration, median, range	139.14 (0–522.59)	n.a.
EDSS, median, range	2.50 (0.00–6.50)	n.a.
Lesion volume, median, range	5.10 (0.12–232.47)	0.09 (0–14.98)

### 2.2 MRI acquisition and preprocessing

All MRI data were acquired on the same 3 T scanner (Tim Trio Siemens, Erlangen, Germany) using a volumetric high-resolution T1 weighted magnetization prepared rapid acquisition gradient echo (MPRAGE) sequence (TR = 1900 ms, TE = 2.55 ms, TI = 900 ms, FOV = 240 × 240 mm^2^, matrix 240 × 240, 176 slices, voxel size: 1 mm isotropic) as well as a volumetric high-resolution fluid-attenuated inversion recovery sequence (FLAIR, TR = 6000 ms, TE = 388 ms, TI = 2100 ms; FOV = 256 × 256 mm^2^, voxel size: 1 mm isotropic). All MR images were bias field corrected using non-parametric non-uniform intensity normalization (Tustison et al., 2010), changed to a robust field of view and linearly oriented to MNI space using FMRIB software tools (Jenkinson and Smith, 2001). The FLAIR images were then co-registered to the MPRAGE images using a spline interpolation with FSL FLIRT (Jenkinson et al., 2002). Lesion segmentation was done semi-automatically on FLAIR using the lesion prediction algorithm (Schmidt, 2017) as implemented in the Lesion Segmentation Toolbox^3^ version 2.0.15. Lesion masks are subsequently manually corrected by two raters using ITK-SNAP (Yushkevich et al., 2006).^4^ Both raters have more than 5 years of experience in T2 lesion segmentation and were supervised by a board-certified neuroradiologist (MS). Raters were not blinded to the diagnosis. Generation of a brain mask and tissue segmentation into gray matter, white matter, and cerebrospinal fluid was achieved using the Computational Anatomy Toolbox version 11.09 (Gaser and Dahnke, 2016) implemented in SPM12 version 7219. The data were preprocessed in that way to ensure that images are in relative realignment while preserving individual structural variations. Only FLAIR data entered the subsequent analyses because this is the most sensitive sequence for lesions and used in clinical routine for diagnosing MS and monitoring disease progression. For computational efficiency initial scan volumes (182 × 218 × 182) were down-sampled to 96 × 114 × 96 voxels (voxel size: 2 mm isotropic) and standardized for each subject using min-max scaling. To analyze what the classifier picks up when there are no lesions, we generated an additional MRI data set, in which the lesions in FLAIR images were filled. For this, we implemented a version of (Valverde et al., 2014), in which lesion areas (according to the manually segmented lesion masks) have been replaced by local average intensities in normal-appearing white matter. White matter maps were obtained from the SPM 12 tissue segmentation algorithm (Ashburner and Friston, 2003).

### 2.3 ADNI data for pre-training

Data used for pre-training were obtained from the Alzheimer's Disease Neuroimaging Initiative (ADNI) database^5^ We have used subjects from ADNI phase 1 who were included in one of two standard MRI collections (Wyman et al., 2013). We only selected MRI data of Alzheimer's disease (AD) patients and cognitive normal subjects, in total 921 MRI scans from 389 subjects (covering one to three time points). Follow-up acquisitions can be interpreted as a form of data augmentation used to increase the variance within the training data base. Demographical information can be found in Table 2. The MRI scans were acquired with 1.5 Tesla scanners at multiple sites and had already undergone gradient non-linearity, intensity inhomogeneity and phantom-based distortion correction. T1-weighted MPRAGE scans were downloaded and warped to MNI space with ANTs (Avants et al., 2011). As for the MS data, the initial scan volumes were down-sampled to 96 × 114 × 96 voxels and standardized.

**Table 2:** Demographics of ADNI data set.

	AD patients	Healthy controls
Subjects [n]	231	158
Female/Male, in %	42% / 58%	48% / 52%
Age (in years), mean ± std	74.98 (± 7.40)	75.93 (± 5.01)

### 2.4 Classification and visualization analyses

Based on the preprocessed FLAIR data, we first trained several CNN models (with and without pre-training, with and without lesion-filling) to discriminate MS patients and healthy controls and then explained the model's decisions for individual subjects in the test data using LRP. For the CNN models, we evaluated the effect of transfer learning by (1) training the model solely on MS data and (2) pre-training the model on ADNI data and fine-tuning it on MS data. To examine whether our pre-trained network can also learn from only normal-appearing brain matter (NABM), i.e. regions without hyperintense lesions, we retrained the network on lesion-filled FLAIR data. As baseline analyses, we included a support vector machine to classify based on (1) lesion volume and (2) preprocessed FLAIR data. Prior to training, the MS data set was randomly split into two sets: (1) a set for training and hyperparameter optimization (85%) and (2) a holdout set used only for final model evaluation (15%). The code for all models and also the lesion filling algorithm is available at https://github.com/derEitel/explainableMS. In the following subsections, we specify our parameter settings for CNNs, transfer learning and visualization techniques (in particular LRP).

#### 2.4.1 Convolutional neural networks

In this study, we used a 3D CNN architecture consisting of four convolutional layers followed by exponential linear units (ELUs) activation functions and four max-pooling layers applied after the first, second and fourth ELU activation. For each convolutional layer, we learned 64 filters with a kernel size of 3 × 3× 3. Finally, a linear layer with an output shape of 1 and a sigmoid activation returns the classification score. To improve generalization, the model has been regularized using a dropout on the outputs of each max-pooling layer (*p* = 0.3), L2-regularization (*λ* = 0.01) using the weights of the third and fourth convolutional layer, and finally early-stopping the training after the validation loss has not improved for 10/15 epochs during pre-training/fine-tuning. We trained all models using the Adam optimizer (Kingma and Ba, 2014). Hyperparameters (including learning rate, L2 regularization and dropout probability) were optimized on 85% of the training data, leaving 15% for validation. After finding suitable hyperparameters, the model performance was tested out-of-sample on the holdout set. To increase robustness, all CNN experiments were repeated 10 times on the same data split, and thus reported metrics are an average over all 10 trials. We report balanced accuracy as a mean between sensitivity and specificity as well as area under the receiver operating characteristic curve (AUC). All code was implemented using Keras (Chollet, 2015) with the TensorFlow (Abadi et al., 2015) backend.^6^

#### 2.4.2 Transfer learning

Due to the small sample size of the MS data set, we employed the principle of transfer learning (Crammer et al., 2008; Duan et al., 2009; Ben-David et al., 2010), which has been shown to improve performance in medical imaging including MRI data (Gupta et al., 2013; Tajbakhsh et al., 2016; Ghafoorian et al., 2017; Hosseini-Asl et al., 2018; Basaia et al., 2019). We pre-trained our CNN model on ADNI MRI data to separate AD patients and healthy controls, and fine-tuned it on the MS data set to separate MS patients and healthy controls. Since the ADNI data set contains multiple scans for several subjects we ensured that validation and testing was done on disjoint subject sets. The average balanced accuracy over all trials was 78.47%. For further analysis, we selected a model from the 10 trials based on its performance, and then picked its training checkpoint with the best validation accuracy of 82.50%. Fine-tuning on the MS data set uses the same model architecture, which is initialized with the weights and biases of the selected pre-trained model instead of randomly distributed values. We allow all layers to re-learn because we transferred a CNN model between rather different tasks and data sets, in particular (1) across diseases (AD to MS) and (2) across MRI sequences (MPRAGE to FLAIR) exhibiting different magnetic field strengths (1.5 and 3 Tesla). Additionally, the data was augmented during fine-tuning, such that during the creation of each mini-batch each image was flipped along the sagittal axis with a probability of 50% and randomly translated between −2 and 2 pixels within the axial plane. We found optimal initial learning rates to be 0.001 in the pre-training and 0.0005 with a 0.002 decay in the fine-tuning phase.

#### 2.4.3 Visualization

Deep learning methods are often criticized for their lack of interpretability and over the last years much research has focused on improving the interpretability of neural networks (Castelvecchi, 2016; Montavon et al., 2018; Lapuschkin et al., 2019). While some work has focused on understanding class representations and functions of individual neurons, others have developed methods to generate heatmaps based on the input data that indicate the importance or relevance of each pixel or voxel for the final classification decision (Bach et al., 2015; Springenberg et al., 2015; Simonyan et al., 2013). The latter approach is in particular promising in the medical field since it allows for explaining in a fast and intuitive way individual classification decisions without the need for delving deeply into the network structure (Böhle et al., 2019). Generally, it is distinguished between local and global attribution methods (Ancona et al., 2017). Whereas local attribution methods represent how a change in a specific voxel would impact the network's output and solely rely on the network's gradient (e.g. sensitivity analysis resulting in image-specific saliency maps), global attribution methods adjust the relevance of the presence of a feature globally by weighting it with the entire input and thus are more suitable for explanation. In the present study, we used LRP, which has been shown to be a powerful global attribution method (Bach et al., 2015; Samek et al., 2017a; Lapuschkin et al., 2019). It uses the classification score *f*(*x*) directly (and not the gradient as in most other visualization methods) and propagates it through the network using the following rule

(1) $$R_{i}=\sum_{j}\frac{x_{i}w_{\mathrm{ji}}}{\sum_{k}x_{k}w_{\mathrm{jk}}+\varepsilon \cdot \mathrm{sign}\left(\sum_{k}x_{k}w_{\mathrm{jk}}\right)}R_{j}.$$

Here, the relevance from layer *R*_*j*_ is propagated to its previous layer *R*_*i*_. The term *ε* is set to a small value (in this study: 0.001) to avoid division by 0. By using both the activation *x* as well as the weights *w* connecting layers *i* and *j*, LRP assigns a larger share to neurons that are more strongly activated and to connections which have been reinforced during training (Samek et al., 2017b). By decomposing the classification score *f*(*x*) rather than the gradient and conserving the classification score during backpropagation, LRP overcomes the flaws of sensitivity analysis (Samek et al., 2017b) and has been shown to provide evidence for AD in individual subjects (Böhle et al., 2019). Recently, it has been shown that LRP can be formulated in the same mathematical framework as other global attribution methods including gradient*input (Shrikumar et al., 2017), integrated gradients (Sundararajan et al., 2017) and DeepLIFT (Shrikumar et al., 2017) and are equivalent under certain assumptions (Ancona et al., 2017).

In this study, we produced individual LRP heatmaps for every subject in the holdout set. We have used the iNNvestigate implementation of LRP (Alber et al., 2018).^7^ For comparison, we produced heatmaps using gradient*input as an alternative global attribution method.

#### 2.4.4 Evaluation of heatmaps

Besides qualitatively comparing individual heatmaps, we compared average heatmaps of MS patients and healthy controls. We evaluated the importance of different brain regions by computing the average relevance for each brain area in the (1) Neuromorphometrics atlas^8^ (Bakker et al., 2015) mostly containing gray matter regions and the (2) JHU DTI-based white-matter atlas^9^ (Mori and Crain, 2005) containing white matter regions. Areas were aggregated between left and right hemisphere and certain substructures are combined into one region. For visualization of (1) we selected the 30 areas with the highest sum of absolute relevance means across MS patients and healthy controls in the test set, yielding areas with both the highest and lowest relevance. Please reconsider here that the MRI data have only been linearly registered and thus slight deviations from the anatomical locations stated in the atlases are conceivable. To evaluate the effect of transfer learning on the heatmaps, we compare average heatmaps for MS patients before and after pre-training. To assess the relevance of normal-appearing brain areas in contrast to lesion areas, we computed relevance scores separately for the original MRI data set and the lesion filled MRI data set. To assess the amount of relevance attributed to the lesions in the original MRI data set, we computed

(2) $$\text{lesion}\;\text{relevance}=\frac{\sum \mathrm{lm}*{\mathrm{hm}}^{+}}{\sum {\mathrm{hm}}^{+}}$$

where *lm* is the individual lesion mask and *hm*^+^ the individual positive relevance.

#### 2.4.5 Baseline analyses

As a baseline we have trained a support vector machine (SVM) to classify between MS patients and healthy controls based on (1) FLAIR lesion load and (2) preprocessed FLAIR volumes. Hyperparameters were tuned on the training data set using grid search, nested within a 5-fold cross-validation (SVM kernel: linear and radial basis function [RBF], *C, γ* = [0.001,0.1,1,10]); for the preprocessed FLAIR volumes an optional prior dimensionality reduction step via principal component analysis was performed.

## 3 Results

### 3.1 Classification performance

In Table 3, we depict the performance for the different classification models. As expected FLAIR lesion load – as one of the core biomarkers in MS – in combination with a SVM led to a high balanced accuracy (88.46%) and a high AUC (94.62%). When instead of the FLAIR lesion load the entire FLAIR volume is used as input to the SVM, the AUC dropped down to 66.92%. The CNN model solely trained on the MS data set resulted in a balanced accuracy of 71.23% and an AUC of 85.46%. When the network has been pre-trained on the ADNI data set and fine-tuned to the MS data set, the balanced accuracy increased by 16 percentage points to 87.04% and is therefore comparable to the performance of the baseline FLAIR lesion load model. Moreover, the pre-trained CNN model outperformed all other classifiers in terms of AUC (96.08%) and importantly also in terms of sensitivity (93.08%). The ROC curve for all 10 trials is shown in supplementary Fig. 1. For further processing we have selected the model with the best validation balanced accuracy from the 10 training repetitions of 91.67%, which achieved a holdout balanced accuracy of 91.15%. Its training curve can be found in supplementary Fig. 2. To assess the impact of normal-appearing brain matter, we trained the same CNN model on lesion-filled FLAIR data. Still, a reasonable balanced accuracy of 70.15% and a relatively high AUC of 90.92% has been achieved.

**Table 3:** Performance (in %) for the different models on the holdout data set. Values are averages over 10 trials. Highest values per column are highlighted in bold. Pre-train., pre-training; Class., classifier; Bal. acc., balanced accuracy; Sens., sensitivity; Spec., specificity; AUC, area under the curve of the receiver operating characteristic; les. fill., lesions filled.

Data	Pre-train.	Class.	Bal. acc.	Sens.	Spec.	AUC
FLAIR lesion load	–	SVM	**88.46%**	76.92%	**100.00**%	94.62%
FLAIR	–	SVM	66.92%	53.85%	80.00%	66.92%
FLAIR	no	CNN	71.23%	68.46%	74.00%	85.46%
FLAIR	yes	CNN	87.04%	**93.08**%	81.00%	**96.08**%
FLAIR - les. fill.	yes	CNN	70.15%	92.31%	48.00%	90.92%

### 3.2 Visualization

**Fig. 2:**
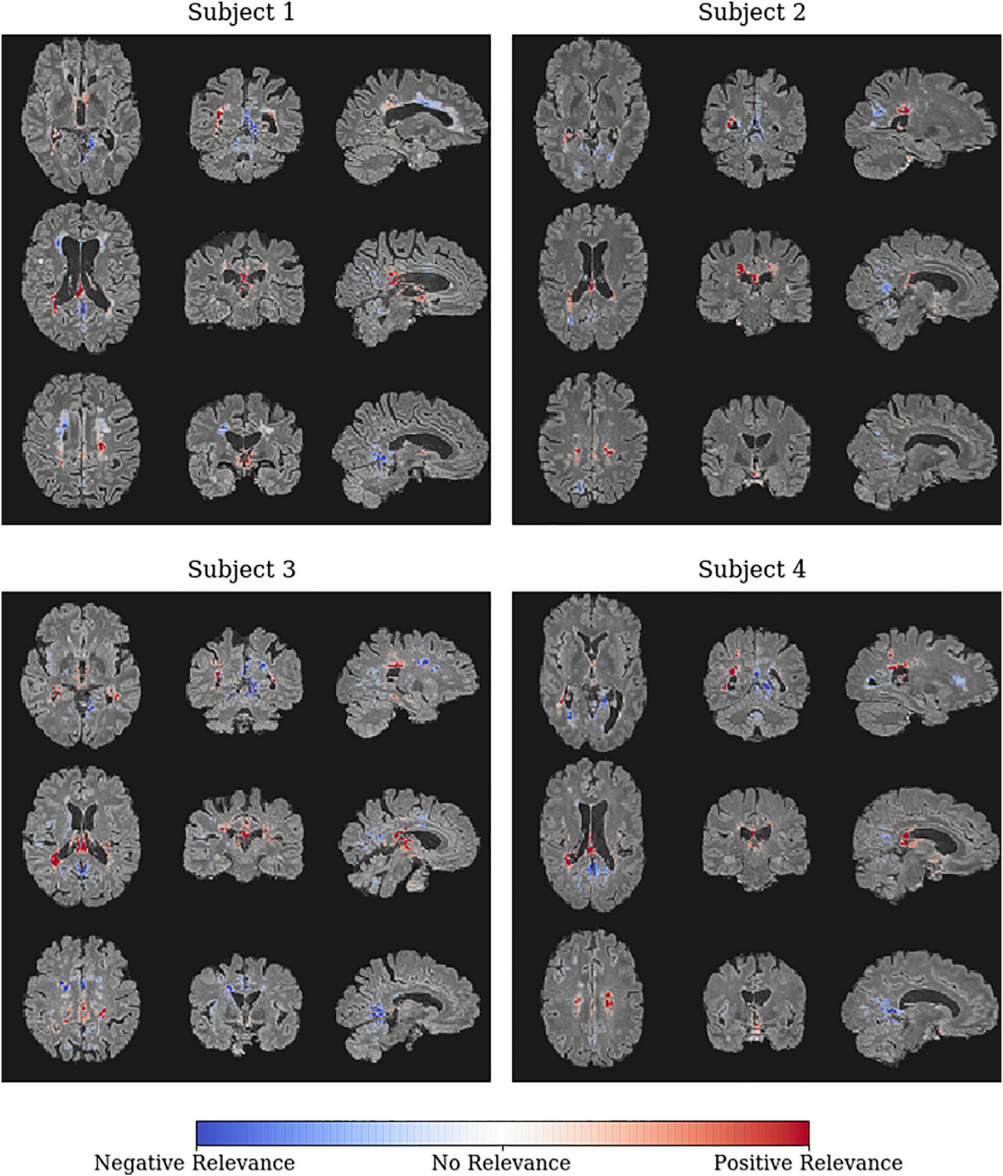
Individual LRP heatmaps (overlayed on the input FLAIR data) for the four MS patients with the highest classification score in terms of the sigmoid output. Heatmap values are normalized in the range [−0.03, 0.03]. Colors indicate regions supporting (red) or rejecting (blue) the classification as a MS patient with respect to the underlying CNN model.

After the CNN models have been trained, we used LRP to generate an individual heatmap for each subject in the holdout data set indicating the relevance of each voxel for the respective classification decision. In Fig. 2, we show the individual heatmaps overlayed on the FLAIR data for four correctly classified MS patients, who achieved the highest classification scores in terms of the sigmoid output. High classification scores generally indicate a higher confidence of the model for the respective classification decision and thus the corresponding explanations are usually more pronounced and less diffuse as for cases with lower classification scores. All four patients have in common that high positive relevance is attributed around the occipital horn of both lateral ventricles and covers periventricular lesion areas as well as the body and splenium of the corpus callosum. Even though the images were clearly classified as MS, certain regions are assigned negative relevance, meaning that these areas speak against the MS diagnosis. Negative relevance can be found around the frontal horn of both ventricles, notably even in periventricular lesion areas (see for example subject 1). Interestingly, lesions not bordering the ventricles seem often to be ignored or are assigned negative relevance. For comparison, we show and discuss individual heatmaps of two misclassified subjects in supplementary Fig. 3.

**Fig. 3:**
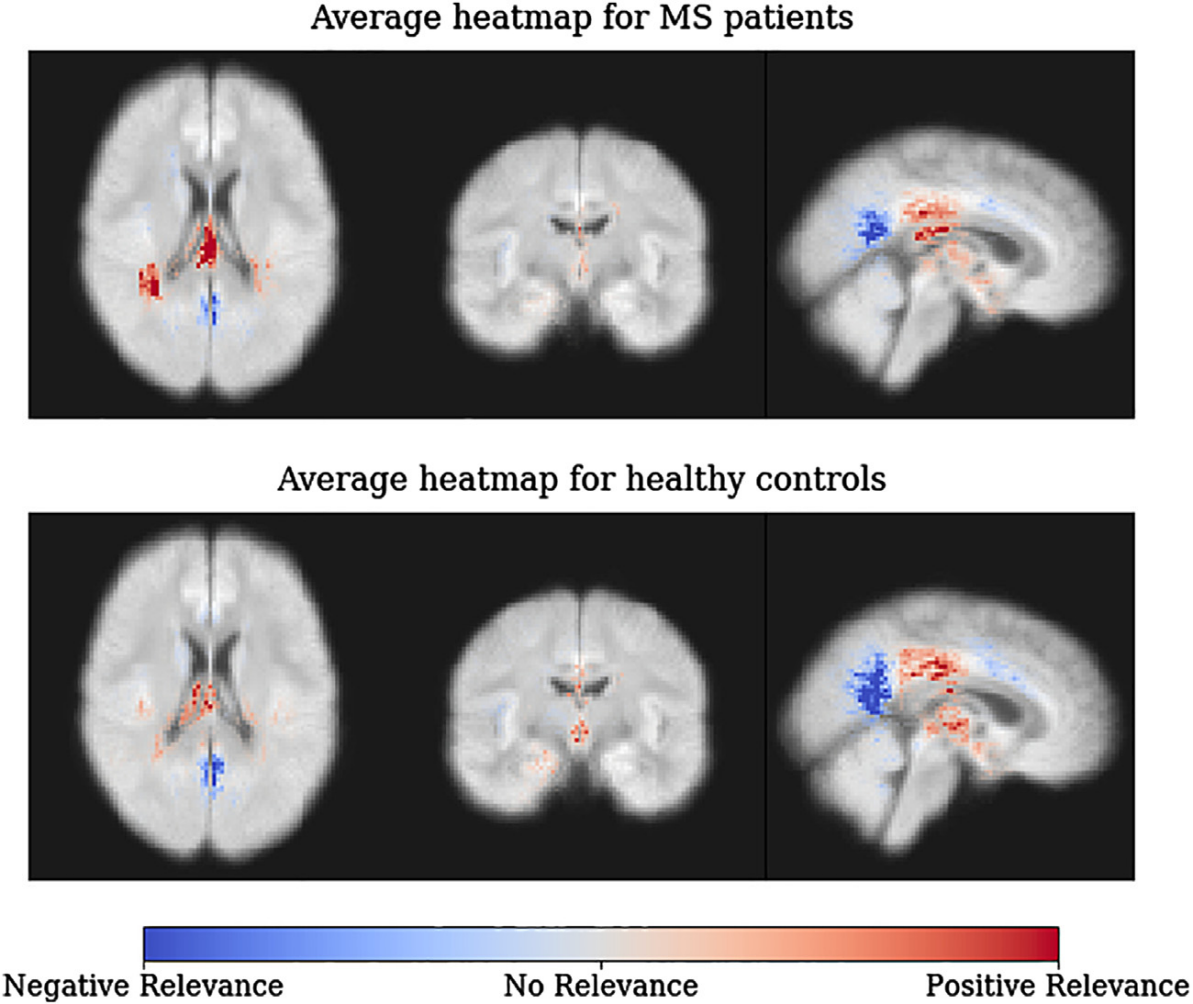
Average LRP heatmaps for all correctly classified MS patients (top) and all correctly classified healthy controls (bottom) in the holdout set. Values are normalized in the range [−0.02, 0.02]. Please note that the underlying brain map has been computed as the average of all training subjects and does not reflect the MRI data of individual subjects.

**Fig. 4:**
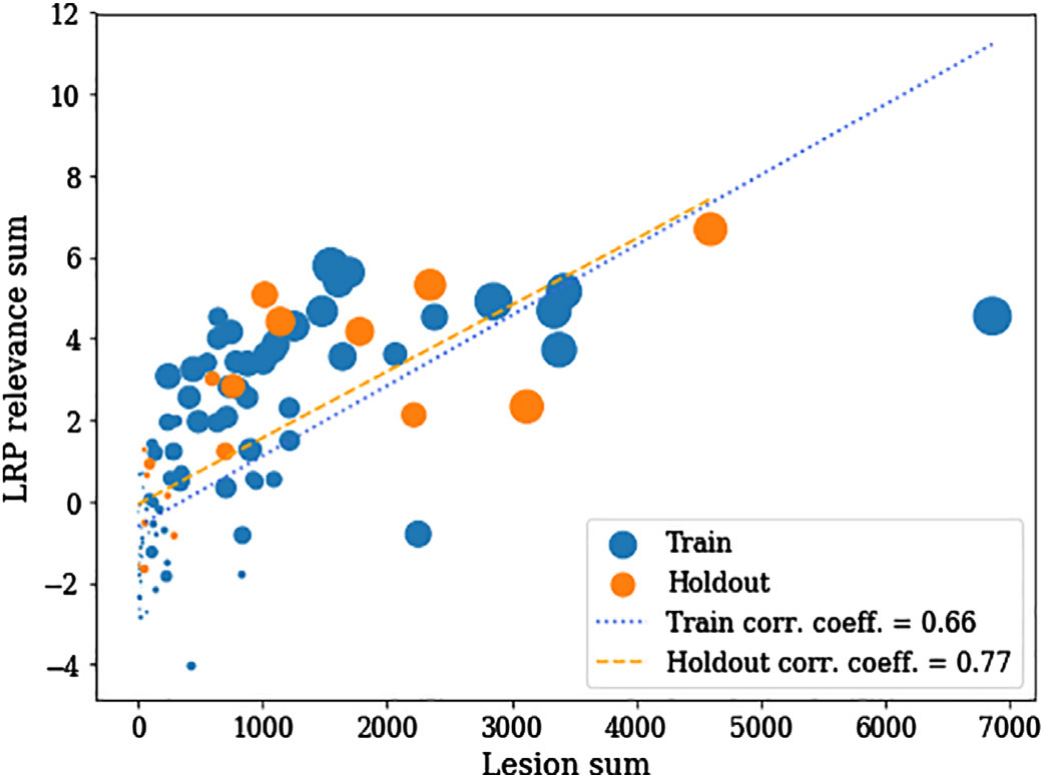
Correlation between lesion sum and LRP relevance sum. The Pearson correlation coefficient is shown for both training and holdout set separately, of which both are significant (*p*_*train*_ < 0.001, *p*_*test*_ < 0.001, permutation test). The size of each data point shows the lesion-relevance similarity according to Eq. (2).

In Fig. 3, we show average heatmaps for all correctly classified MS patients (top) and all correctly classified healthy controls (bottom) in the holdout set. In accordance with the heatmaps of the individual subjects in Fig. 2, posterior periventricular white matter regions have a strong positive relevance for the MS diagnosis. This is true for both MS patients and healthy controls, but the effect is less pronounced for healthy controls. The reversed effect can be seen for clusters exhibiting negative relevance in white matter areas in the corpus callosum and close to occipital and parietal lobe. Over all voxels healthy controls typically obtain a negative relevance sum (mean ± std.: −1.05e-6 ± 0.0013) as opposed to a positive relevance sum in MS patients (3.07e-06 ± 0.0014). Notably, the total relevance attributed to lesion areas was on average 5.15% (on MS patients 9.71%) compared to a lesion coverage of only 0.41% in the training data set. In Fig. 4, we show that the sum of voxels containing lesions (referred to as lesion sum) and LRP relevance sum are significantly correlated for training and hold-out data.

**Fig. 5:**
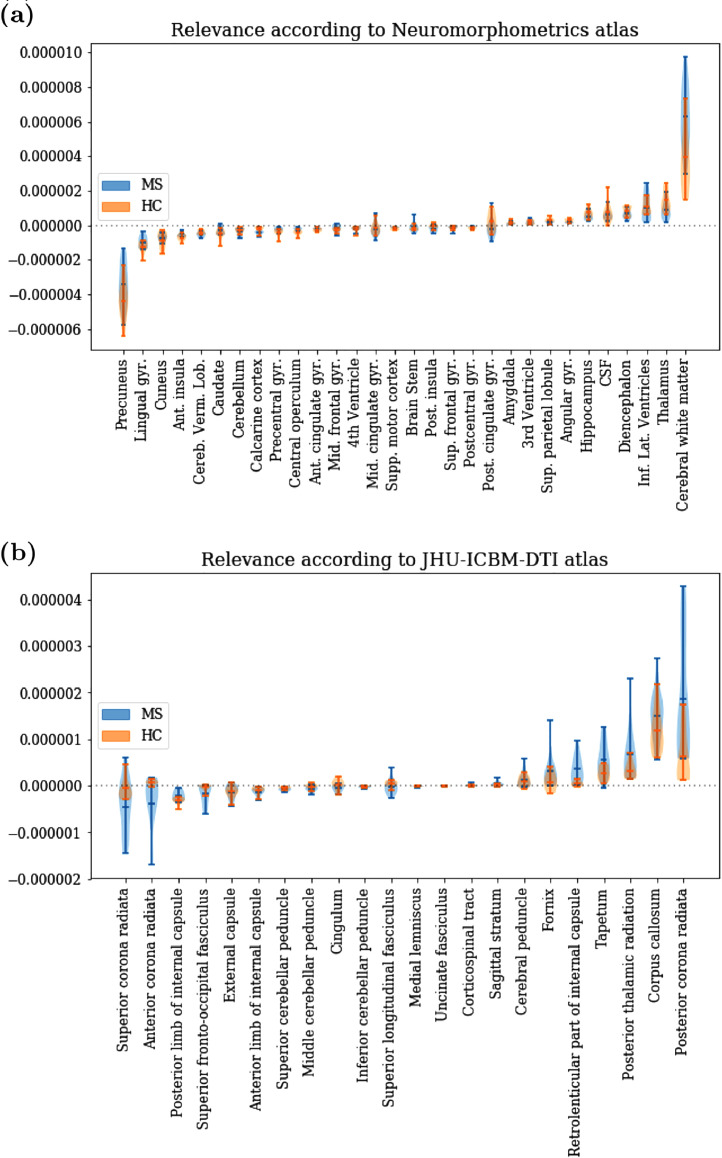
LRP relevance distribution over (a) 30 (mainly) gray matter areas from the Neuromorphometrics atlas and (b) 22 white matter areas from the JHU ICBM-DTI atlas, separately for MS patients and healthy controls in the holdout set. The absolute values per region are rather small as LRP aims to conserve the sigmoid output and distributes it over all voxels.

In Fig. 5, we depict the region-wise LRP relevance for MS diagnosis, separately for MS patients and healthy controls. In the Neuromorphometrics atlas (see Fig. 5a), most relevance is attributed to cerebral white matter, followed by thalamus, lateral ventricles and diencephalon. Negative relevance is strongest in the precuneus, followed by lingual gyrus, cuneus and insula. In the JHU white matter atlas (see Fig. 5b), most positive relevance is attributed to posterior corona radiata and corpus callosum, followed by posterior thalamic radiation, tapetum, internal capsule and fornix. Notably, these areas are generally characterized by a high lesion density, which is also present in this MS data set (see supplementary Figs. 4 and 5). Negative relevance has been found in the superior and anterior corona radiata. Generally, the relevance for MS patients is higher in white matter than in gray matter areas. Moreover, the differences between MS patients and healthy controls are more pronounced in white matter areas.

The qualitative and quantitative analysis using another global attribution method, namely gradient*input, produced highly similar results as shown in supplementary Figs. 6 and 7.

**Fig. 6:**
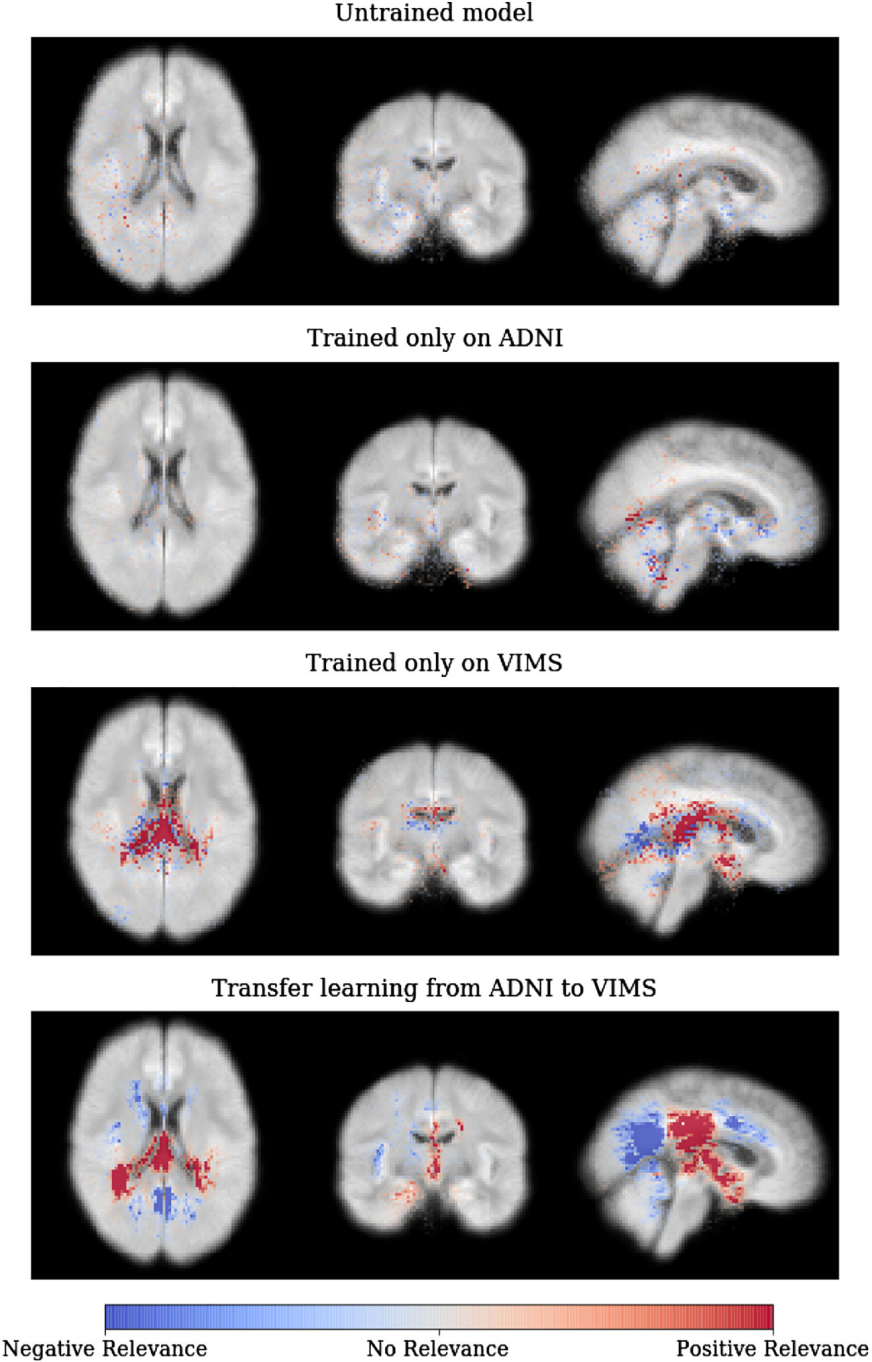
Average heatmaps for different CNN models applied to the MS (VIMS) cohort – starting from an untrained CNN model with random parameters over a CNN trained only on either ADNI or MS data to a CNN pre-trained on ADNI and fine-tuned on MS. As it can be seen, the fine-tuned model led to the most concise regions of positive and negative relevance. Please note that we averaged here the heatmaps over all (not only the correctly classified) MS patients in the holdout set and that the heatmap values here are not normalized to a fixed range but shown with respect to the minimum value of the untrained model.

In Fig. 6, we show the effects of transfer learning on the average relevance heatmaps for the MS patients in the holdout set. For the untrained model with random parameters (first row), only scarcely distributed individual voxels attain tiny relevance values. For the CNN model trained on ADNI and directly applied to MS patients (without fine-tuning; second row), more voxels are attributed relevance and are diffusely clustered. For the CNN model trained only on MS data (without pre-training; third row), strong relevance is projected to the ventricles and periventricular white matter. And finally, for the pre-trained model (transfer learning from ADNI to MS; last row), distinct clusters for both positive and negative relevance can be detected, which are more delineated than for the CNN model without pre-training.

**Fig. 7:**
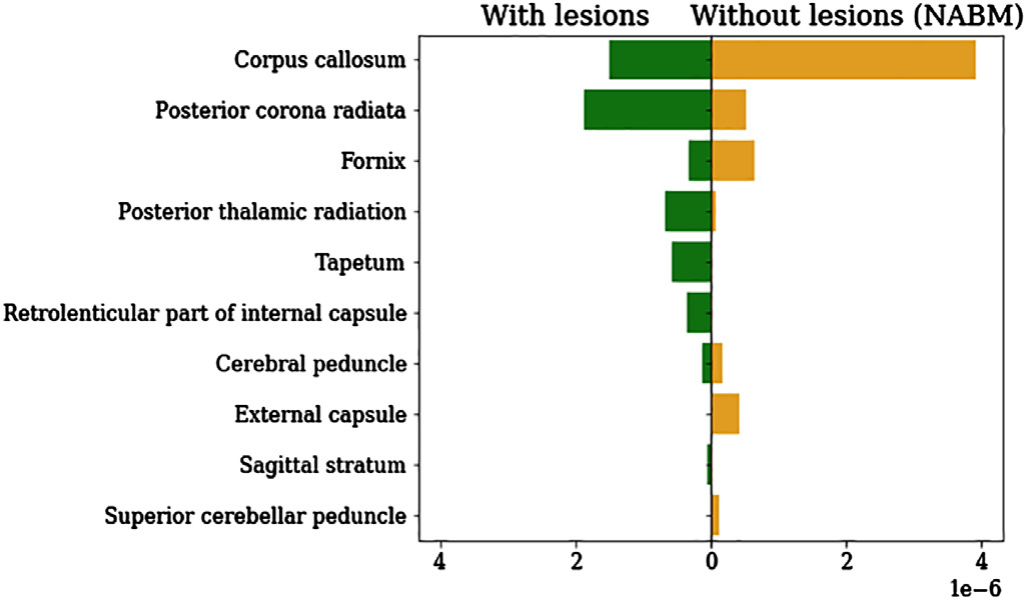
Comparison of average relevance distribution over white matter areas for a CNN model trained on original FLAIR data (left) and lesion-filled FLAIR data (right; NABM, normal-appearing brain matter). We calculated the relevance sum of both models (averaged over subjects) and show the 10 areas with the highest score.

To assess the contribution of normal-appearing brain matter, we compared the relevance maps between the CNN models trained on the original FLAIR data and the lesion-filled FLAIR data (for the performance see Table 3). In Fig. 7, we depict the relevance for the 10 top-scored white matter regions, separately for both models. In general one can see that the relevance shifts from a distribution more evenly spread among multiple areas to a distribution with a prominent peak and otherwise low shares of relevance. Notably, relevance is shifted away from areas with large amounts of lesions such as posterior corona radiata, posterior thalamic radiata as well as tapetum towards mainly the corpus callosum and regions with very few lesions like fornix and external capsule (see supplementary Fig. 4 for distribution of white matter lesions).

## 4 Discussion

### 4.1 Summary

In the present study, we introduced a transparent framework for analyzing neuroimaging data with CNNs that is able to explain individual classification decisions. By utilizing transfer learning we could further achieve good classification results from only a small data set of task-specific data. In combination with LRP, we could demonstrate the capacity of our framework to learn significant MS-relevant information from conventional MRI data. Notably, a pre-trained CNN was able to identify MS patients with an accuracy similar to a classical machine learning analysis, in which the FLAIR lesion load was used as input. This is quite remarkable, because the CNN model was considered to be naive by not being provided with any prior information on MS-relevant features such as hyperintense lesions. The subsequent visualization analysis, using heatmaps generated by LRP, revealed that the CNN model indeed uses (posterior) white matter lesions as primary information source. In addition, other information, e.g. in normal-appearing white and gray matter (e.g. the thalamus) have been found useful by the CNN model.

### 4.2 Related work

Compared to other neurological diseases, in particular AD, only a few MS studies exist that employ machine learning methods outside the scope of lesion segmentation. We think that the main reasons are (1) the lack of easy accessible large open data bases such as the Alzheimer's Neuroimaging Initiative (ADNI) data base and (2) the focus on white matter lesion volume as primary MRI-derived outcome measure in MS. Classical machine learning methods in combination with more or less sophisticated feature extraction methods, from both conventional and advanced MRI data, have been used to (1) diagnose MS (Weygandt et al., 2011; Hackmack et al., 2012b; Zurita et al., 2018; Eshaghi et al., 2016) (2) decode symptom severity (Hackmack et al., 2012a) (3) identify clinical subtypes (Eshaghi et al., 2018; Nichols et al., 2012; Eshaghi et al., 2015) and (4) predict conversion from clinically isolated syndrome to MS (Wottschel et al., 2015; Bendfeldt et al., 2019). Deep learning architectures have so far been implemented for lesion segmentation (Valverde et al., 2017; Li et al., 2016; Khastavaneh and Ebrahimpour-Komleh, 2017), predicting MS based on binary lesion masks (Yoo et al., 2016), modelling brain and lesion variability (Brosch, 2016) and finding differences in normal-appearing brain matter based on T1-weighted and myelin images (Yoo et al., 2018). To the best of our best knowledge, the present study is the first study employing CNNs and advanced visualization techniques for diagnosing MS based on the clinically most relevant MRI sequence (i.e. FLAIR).

It is generally recognized that, especially in the medical field, it is very important that classification decisions are reasonably explained even in light of high accuracies (which are no guarantee for a – from a human perspective – sensible discrimination strategy (Lapuschkin et al., 2019; Lapuschkin et al., 2016)). Although a number of methods exist that generate individual heatmaps (Zeiler and Fergus, 2014; Springenberg et al., 2015; Simonyan et al., 2013; Zintgraf et al., 2017), we focused here on the LRP method (Bach et al., 2015; Montavon et al., 2018; Lapuschkin et al., 2019) which has a solid theoretical framework and has been extensively validated (see e.g. (Samek et al., 2017a; Lapuschkin et al., 2019; Samek et al., 2017b)). Very recently, LRP has shown to be very helpful for explaining cognitive states or AD diagnosis in deep neural networks trained on either functional or structural MRI data (Böhle et al., 2019; Thomas et al., 2018). To the best of our knowledge, these are the only applications of LRP in the neuroimaging field. In the present study, we demonstrated that LRP is capable of identifying reasonable areas supporting a MS diagnosis in addition to features needing further clinical validation. Those areas have been shown to be robust using gradient*input as a different visualization method. By this, we have shown that those heatmaps can be very valuable in explaining decisions of neural networks trained on small sample sizes and to verify whether an algorithm has learned something meaningful (i.e. matching domain knowledge) or just spotted biases or artifacts in the data (see also (Springenberg et al., 2015; Lapuschkin et al., 2019)).

### 4.3 Key findings

#### 4.3.1 CNNs learn to identify lesions as an important biomarker for MS

Although our pre-trained CNN model did not get any prior information about the relevance of hyperintense lesions for MS, it learned to successfully identify lesions as a primary information source. Notably, the total relevance attributed to lesion areas was on average 5.15% (on MS patients 9.71%) compared to a lesion coverage of only 0.41% in the training data set. In addition, LRP relevance sum was significantly correlated to lesion sum. We show that LRP heatmaps not only detect single lesions in individual patients but generally attributed most positive relevance to white matter areas around the posterior occipital horns. Importantly, the CNN model did not simply assign high relevance to hyperintense areas in the brain, but learned to distinguish between different lesion locations: while anterior periventricular lesions as well as lesions not bordering the lateral ventricles were assigned no or negative relevance, only posterior periventricular lesion areas were assigned positive relevance for MS. Interestingly, hyperintensities in posterior ventricular regions seem to be the main reason why the healthy control in supplementary Fig. 3 has been misclassified as MS patient. In general, strongest positive relevance was found in posterior corona radiata, corpus callosum and thalamic radiation, which are characterized by a high lesion density in MS patients (see (Gass et al., 2012) and supplementary Figs. 4 and 5).

#### 4.3.2 CNNs learn to identify relevant areas beyond lesions

The CNN model primarily focuses on lesions, but relevance has also been attributed to gray matter areas such as the thalamus, which is known to be affected in MS from earliest disease stages (Azevedo et al., 2018; Azevedo et al., 2015). To further investigate what the CNN model learns beyond lesions, we repeated the analysis on lesion filled FLAIR data. As expected, the balanced accuracy as well as AUC decreased (by almost 17 and 6 percentage points respectively) and relevance has shifted away from regions which typically contain hyperintense lesions. The region that was assigned most relevance after lesion removal was the corpus callosum. While the corpus callosum is generally susceptible to demyelinating lesions (Barnard and Triggs, 1974; Garg et al., 2015; Renard et al., 2014) the literature also suggests further biomarkers such as axonal loss and diffuse atrophy (Renard et al., 2014; Evangelou et al., 2000) or narrow T2 hyperintense bands along the callosal-septal interface (Garg et al., 2015). The fornix, even though it contains a very small amount of lesions (see supplementary Fig. 4 and (Thomas et al., 2011)), is assigned positive relevance with lesions and an increased relevance without lesions. It has been shown that lower fractional anisotropy in the fornix is exhibited in MS subjects in comparison to healthy controls (Roosendaal et al., 2009; Kern et al., 2012). Additionally, external capsule and superior cerebellar peduncle receive only positive relevance after lesion removal, which were found to be affected in MS patients (Anderson et al., 2011; Zhang et al., 2017). These results are generally in line with other machine learning studies finding differences in normal-appearing brain matter in MS patients (Weygandt et al., 2011; Hackmack et al., 2012a; Yoo et al., 2018). It would be very interesting to further investigate whether our findings correlate with underlying pathological mechanisms only demonstrable by advanced MRI sequences such as diffusion weighted imaging or magnetization transfer imaging.

#### 4.3.3 Transfer learning improves learning across diseases and MRI sequences

In recent years, transfer learning has been successfully employed in brain lesion segmentation (Ghafoorian et al., 2017) and AD classification (Gupta et al., 2013; Hosseini-Asl et al., 2018; Payan and Montana, 2015). The latter studies used either autoencoders trained on MRI data or natural images (Gupta et al., 2013; Payan and Montana, 2015) or used one AD data set for pre-training and another AD data set for fine-tuning (Hosseini-Asl et al., 2018). In the present study, we have shown that transfer learning can also help in learning (1) across diseases (AD to MS) and (2) across MRI sequences (MPRAGE to FLAIR) exhibiting different magnetic field strengths (1.5 and 3 Tesla). We demonstrated that not only the balanced accuracy increases drastically (about 16 percentage points), but also that LRP leads to much more focused heatmaps concentrating on (posterior) periventricular lesion areas. Given that our pre-trained model performed similar to a classical machine learning analysis using FLAIR lesion load as a classical biomarker in MS, we believe that larger data sets might allow for outperforming models based on lesion masks in the future. Additionally, we are convinced that our approach – given a reasonable data basis – might also be very useful in answering more complex questions such as predicting disease progression.

### 4.4 Limitations

The main limitation of this study is the limited sample size. Although a sample size of *n* = 147 is comparable with other deep learning studies in the neuroimaging field (Vieira et al., 2017), it is generally considered to be too low to learn robust representations from the data and to generalize to other data sets. To partly alleviate this problem, we pre-trained our network on ADNI data (*n* = 921) and fine-tuned it on the MS data. By visualizing the average heatmaps for MS patients, we show in addition to a balanced accuracy of 87.04 % that the CNN captures MS-relevant information by focusing on posterior ventricular regions usually characterized by a high rate of MS lesion incidences. Nevertheless, future studies should verify our results in larger data sets, preferably coming from different sites. Another limitation, related to the first one, is that we were limited in the choice of architecture used for the CNN analysis. Very deep networks with a high capacity easily overfit on data sets with less than hundreds or thousands of samples per class. Furthermore, since we use volumetric data the additional dimension as compared to 2D images causes each layer to consume substantially more GPU memory, which makes it a strongly limiting factor in architecture design. However, we found a relatively simple CNN architecture to be successful together with several regularization methods (drop out, L2-regularization and early stopping). Moreover, by registering the MRI data only linearly to MNI space, the regions contained in both atlases only roughly correspond to individual anatomical locations. On the other hand, non-linear registration can lead to strong deformations, in particular in patients, and we show here that our CNN model can also operate on a more native level (in accordance with (Suk et al., 2014)). To be able to make more specific anatomical claims in individual subjects, future studies might use individual atlases. And finally, heatmaps do neither allow to determine the underlying pathological mechanism (e.g. atrophy, demyelination or axonal loss) resulting in assigning a voxel to be relevant or to assess interactions between voxels. For this, one would have to take a deeper look into the specific filters that have been learned throughout the training process in combination with MR sequences more sensitive for certain tissue damage (e.g. diffusion weighted or myelin imaging). Nevertheless, we still believe that heatmaps can be very helpful in supplementing individual disease diagnoses by providing a simple and intuitive explanation.

## 5 Conclusion

In conclusion, we have shown that our framework helps in uncovering CNN decisions for diagnosing MS based on FLAIR data using LRP. In particular, we demonstrated that (1) CNN models pre-trained on AD data are capable of successfully separating MS patients and controls on a typically sized neuroimaging cohort and (2) LRP is not only very valuable in explaining individual network's decisions, but also in generally helping to assess whether CNN models have learned significant features. Notably, our CNN models focus on hyperintense lesions as primary information source, but also incorporates information from lesion location and normal-appearing brain areas. We see a high potential in the combination of CNNs, transfer learning and LRP heatmaps and are convinced that our framework might not only be helpful in other disease decoding studies, but also for answering more complex questions such as predicting disease progression or treatment response in individual subjects.

## Funding

We acknowledge support from the German Research Foundation (DFG, 389563835), the Manfred and Ursula-Müller Stiftung and Charité – Universitätsmedizin Berlin (Rahel-Hirsch scholarship and Open Access Publication Fund).

## Appendix A Supplementary data

Supplementary material Image 1

## Footnotes

2 https://neurocure.de/en/clinical-center/clinical-studies/current-studies.html

3 http://www.statistical-modelling.de/lst.html

4 http://www.itksnap.org

5 http://adni.loni.usc.edu, RRID:SCR_003007

6 Keras version 2.2; TensorFlow version 1.11

7 The implementation can be found at https://github.com/albermax/innvestigate

8 Contained in the SPM12 software, https://www.fil.ion.ucl.ac.uk/spm/software/spm12/

9 https://fsl.fmrib.ox.ac.uk/fsl/fslwiki/Atlases

## References

[bb0005] Abadi M. (2015). TensorFlow: Large-Scale Machine Learning on Heterogeneous Systems.

[bb0010] Absinta M., Sati P., Reich D.S. (2016). Advanced MRI and staging of multiple sclerosis lesions. Nat. Rev. Neurol..

[bb0015] Alber M., Lapuschkin S., Seegerer P., Hägele M., Schütt K.T., Montavon G., Samek W., Müller K.-R., Dähne S., Kindermans P.-J. (2018). iNNvestigate neural networks!. CoRR abs/1808.

[bb0020] Ancona M., Ceolini E., Öztireli A.C., Gross M.H. (2017). A unified view of gradient based attribution methods for deep neural networks. CoRR abs/1711.

[bb0025] Anderson V.M., Wheeler-Kingshott C.A., Abdel-Aziz K., Miller D.H., Toosy A., Thompson A.J., Ciccarelli O. (2011). A comprehensive assessment of cerebellar damage in multiple sclerosis using diffusion tractography and volumetric analysis. Mult. Scler. J..

[bb0030] Ashburner J., Friston K.J., Frackowiak R.S.J., Friston K.J., Frith C., Dolan R., Friston K.J., Price C.J., Penny W.D. (2003). Image segmentation. Human Brain Function.

[bb0035] Avants B.B., Tustison N.J., Song G., Cook P.A., Klein A., Gee C. (2011). A reproducible evaluation of ANTs similarity metric performance in brain image registration. Neuroimage.

[bb0040] Azevedo C.J., Overton E., Khadka S., Buckley J., Liu S., Sampat M., Kantarci O., Frenay C.L., Siva A., Okuda D.T. (2015). Early cns neurodegeneration in radiologically isolated syndrome. Neurol. Neuroimmunol. Neuroinammat..

[bb0045] Azevedo C.J., Cen S.Y., Khadka S., Liu S., Kornak J., Shi Y., Zheng L., Hauser S.L., Pelletier D. (2018). Thalamic atrophy in multiple sclerosis: a magnetic resonance imaging marker of neurodegeneration throughout disease. Ann. Neurol..

[bb0050] Bach S., Binder A., Montavon G., Klauschen F., Müller K.-R., Samek W. (2015). On pixel-wise explanations for non-linear classifier decisions by layer-wise relevance propagation. PLoS One.

[bb0055] Backner Y., Kuchling J., Massarwa S., Oberwahrenbrock T., Finke C., Bellmann-Strobl J., Ruprecht K., Brandt A.U., Zimmermann H., Raz N., Paul F., Levin N. (2018). Anatomical wiring and functional networking changes in the visual system following optic neuritis. JAMA Neurol..

[bb0060] Bakker R., Tiesinga P., Kötter R. (2015). The scalable brain atlas: instant web-based access to public brain atlases and related content. Neuroinformatics.

[bb0065] Barnard R.O., Triggs M. (1974). Corpus callosum in multiple sclerosis. J. Neurol. Neurosurg. Psychiatry.

[bb0070] Basaia S., Agosta F., Wagner L., Canu E., Magnani G., Santangelo R., Filippi M. (2019). Automated classification of Alzheimer's disease and mild cognitive impairment using a single MRI and deep neural networks. NeuroImage.

[bb0075] Ben-David S., Blitzer J., Crammer K., Kulesza A., Pereira F., Vaughan J.W. (2010). A theory of learning from different domains. Mach. Learn..

[bb0080] Bendfeldt K., Taschler B., Gaetano L., Madoerin P., Kuster P., Mueller-Lenke N., Amann M., Vrenken H., Wottschel V., Barkhof F., Borgwardt S., Klöppel S., Wicklein E.-M., Kappos L., Edan G., Freedman M.S., Montalbán X., Hartung H.-P., Pohl C., Sandbrink R., Sprenger T., Radue E.-W., Wuerfel J., Nichols T.E. (2019). Mri-based prediction of conversion from clinically isolated syndrome to clinically definite multiple sclerosis using svm and lesion geometry. Brain Imag. Behav..

[bb0085] Böhle M., Eitel F., Weygandt M., Ritter K. (2019). Layer-wise relevance propagation for explaining deep neural network decisions in mri-based alzheimer's disease classification. Front. Aging Neurosci..

[bb0090] Brosch T. (2016). Efficient Deep Learning of 3D Structural Brain MRIs for Manifold Learning and Lesion Segmentation with Application to Multiple Sclerosis.

[bb0095] Castelvecchi D. (2016). Can we open the black box of AI?. Nature.

[bb0100] Chollet F. (2015). Keras. https://github.com/fchollet/keras.

[bb0105] Crammer K., Kearns M., Wortman J. (2008). Learning from multiple sources. J. Mach. Learn. Res..

[bb0110] De Fauw J., Ledsam J.R., Romera-Paredes B., Nikolov S., Tomasev N., Blackwell S., Askham H., Glorot X., O'Donoghue B., Visentin D., van den Driessche G., Lakshminarayanan B., Meyer C., Mackinder F., Bouton S., Ayoub K., Chopra R., King D., Karthikesalingam A., Hughes C.O., Raine R., Hughes J., Sim D.A., Egan C., Tufail A., Montgomery H., Hassabis D., Rees G., Back T., Khaw P.T., Suleyman M., Cornebise J., Keane P.A., Ronneberger O. (2018). Clinically applicable deep learning for diagnosis and referral in retinal disease. Nat. Med..

[bb0115] Duan L., Tsang I.W., Xu D., Chua T.-S. (2009). Domain adaptation from multiple sources via auxiliary classifiers. Proceedings of the 26th Annual International Conference on Machine Learning - ICML ‘09.

[bb0120] Eshaghi A., Riyahi-Alam S., Saeedi R., Roostaei T., Nazeri A., Aghsaei A., Doosti R., Ganjgahi H., Bodini B., Shakourirad A., Pakravan M., Ghanaati H., Firouznia K., Zarei M., Azimi A.R., Sahraian M.A. (2015). Classification algorithms with multi-modal data fusion could accurately distinguish neuromyelitis optica from multiple sclerosis. NeuroImage.

[bb0125] Eshaghi A., Wottschel V., Cortese R., Calabrese M., Sahraian M.A., Thompson A.J., Alexander D.C., Ciccarelli O. (2016). Gray matter mri differentiates neuromyelitis optica from multiple sclerosis using random forest. Neurology.

[bb0130] Eshaghi A., Marinescu R.V., Young A.L., Firth N.C., Prados F., Jorge Cardoso M., Tur C., De Angelis F., Cawley N., Brownlee W.J., De Stefano N., Laura Stromillo M., Battaglini M., Ruggieri S., Gasperini C., Filippi M., Rocca M.A., Rovira A., Sastre-Garriga J., Geurts J.J., Vrenken H., Wottschel V., Leurs C.E., Uitdehaag B., Pirpamer L., Enzinger C., Ourselin S., Gandini Wheeler-Kingshott C.A., Chard D., Thompson A.J., Barkhof F., Alexander D.C., Ciccarelli O. (2018). Progression of regional grey matter atrophy in multiple sclerosis. Brain.

[bb0135] Evangelou N., Konz D., Esiri M.M., Smith S., Palace J., Matthews P.M. (2000). Regional axonal loss in the corpus callosum correlates with cerebral white matter lesion volume and distribution in multiple sclerosis. Brain.

[bb0140] Filippi M., Rocca M.A., Ciccarelli O., De Stefano N., Evangelou N., Kappos L., Rovira A., Sastre-Garriga J., Tintoré M., Frederiksen J.L., Gasperini C., Palace J., Reich D.S., Banwell B., Montalban X., Barkhof F. (2016). MRI criteria for the diagnosis of multiple sclerosis: MAGNIMS consensus guidelines. Lancet Neurol..

[bb0145] Garg N., Reddel S.W., Miller D.H., Chataway J., Riminton D.S., Barnett Y., Masters L., Barnett M.H., Hardy T.A. (2015). The corpus callosum in the diagnosis of multiple sclerosis and other CNS demyelinating and inammatory diseases. J. Neurol. Neurosurg. Psychiatry.

[bb0150] Gaser C., Dahnke R. (2016). Cat-a computational anatomy toolbox for the analysis of structural mri data. HBM.

[bb0155] Gass A., Radue E.-W., Nichols T.E., Barkhof F., Vrenken H., Traud S., Kappos L., Polman C., Naegelin Y., Sprenger T., Kuster P., Bendfeldt K., Mueller-Lenke N., Filli L., Hofstetter L., Borgwardt S.J. (2012). Spatiotemporal distribution of white matter lesions in relapsingremitting and secondary progressive multiple sclerosis. Mult. Scler. J..

[bb0160] Geraldes R., Ciccarelli O., Barkhof F., De Stefano N., Enzinger C., Filippi M., Hofer M., Paul F., Preziosa P., Rovira A., DeLuca G.C., Kappos L., Yousry T., Fazekas F., Frederiksen J., Gasperini C., Sastre-Garriga J., Evangelou N., Palace J. (2018). The current role of MRI in differentiating multiple sclerosis from its imaging mimics. Nat. Rev. Neurol..

[bb0165] Ghafoorian M., Mehrtash A., Kapur T., Karssemeijer N., Marchiori E., Pesteie M., Guttmann C.R.G., de Leeuw F.-E., Tempany C.M., van Ginneken B., Fedorov A., Abolmaesumi P., Platel B., Wells W.M., Descoteaux M., Maier-Hein L., Franz A., Jannin P., Collins D.L., Duchesne S. (2017). Transfer learning for domain adaptation in MRI: application in brain lesion segmentation. Medical Image Computing and Computer Assisted Intervention - MICCAI.

[bb0170] Gupta A., Ayhan M., Maida A., Dasgupta S., Mcallester D. (2013). Natural image bases to represent neuroimaging data. Proceedings of the 30th International Conference on Machine Learning (ICML-13).

[bb0175] Hackmack K., Weygandt M., Pfueller C.F., Bellmann-Strobl J., Wuerfel J., Haynes J.-D., Paul F. (2012). Can we overcome the clinico-radiological paradox’ in multiple sclerosis?. J. Neurol..

[bb0180] Hackmack K., Paul F., Weygandt M., Allefeld C., Haynes J.D. (2012). Multiscale classification of disease using structural MRI and wavelet transform. NeuroImage.

[bb0185] Hosseini-Asl E., Ghazal M., Mahmoud A., Aslantas A., Shalaby A.M., Casanova M.F., Barnes G.N., Gimel'farb G., Keynton R., El-Baz A. (2018). Alzheimer's disease diagnostics by a 3D deeply supervised adaptable convolutional network. Front. Biosci..

[bb0190] Jenkinson M., Smith S. (2001). A global optimisation method for robust affine registration of brain images. Med. Image Anal..

[bb0195] Jenkinson M., Bannister P., Brady M., Smith S. (2002). Improved optimization for the robust and accurate linear registration and motion correction of brain images. Neuroimage.

[bb0200] Kern K.C., Ekstrom A.D., Suthana N.A., Giesser B.S., Montag M., Arshanapalli A., Bookheimer S.Y., Sicotte N.L. (2012). Fornix damage limits verbal memory functional compensation in multiple sclerosis. NeuroImage.

[bb0205] Khastavaneh H., Ebrahimpour-Komleh H. (2017). Neural network-based learning kernel for automatic segmentation of multiple sclerosis lesions on magnetic resonance images. J. Biomed. Phys. Eng..

[bb0210] Kingma D.P., Ba J. (2014). Adam: A Method for Stochastic Optimization. http://arxiv.org/abs/1412.6980.

[bb0215] Kuchling J., Backner Y., Oertel F.C., Raz N., Bellmann-Strobl J., Ruprecht K., Paul F., Levin N., Brandt A.U., Scheel M. (2018). Comparison of probabilistic tractography and tract-based spatial statistics for assessing optic radiation damage in patients with autoimmune inammatory disorders of the central nervous system. NeuroImage.

[bb0220] Lapuschkin S., Binder A., Montavon G., Muller K.-R., Samek W. (2016). Analyzing Classifiers: Fisher Vectors and Deep Neural Networks.

[bb0225] Lapuschkin S., Wäldchen S., Binder A., Montavon G., Samek W., Müller K.-R. (2019). Unmasking clever Hans predictors and assessing what machines really learn. Nat. Commun..

[bb0230] Lecun Y., Bengio Y., Hinton G. (2015). Deep learning. Nature.

[bb0235] Li D.K.B., Brosch T., Tang L.Y.W., Traboulsee A., Tam R., Yoo Y. (2016). Deep 3D convolutional encoder networks with shortcuts for multiscale feature integration applied to multiple sclerosis lesion segmentation. IEEE Trans. Med. Imaging.

[bb0240] Litjens G., Kooi T., Bejnordi B.E., Setio A.A.A., Ciompi F., Ghafoorian M., van der Laak J.A.W.M., van Ginneken B., Sánchez C.I. (2017). A Survey on Deep Learning in Medical Image Analysis. http://arxiv.org/abs/1702.05747.

[bb0245] Lowe M.J., Phillips M.D., Lurito J.T., Mattson D., Dzemidzic M., Mathews V.P. (2002). Multiple sclerosis: low-frequency temporal blood oxygen level dependent fluctuations indicate reduced functional connectivity – initial results. Radiology.

[bb0250] Mitchell T., Culpepper W.J., Nichols E., Bhutta Z.A., Gebrehiwot T.T., Hay S.I., Khalil I.A., Krohn K.J., Liang X., Naghavi M., Mokdad A.H., Nixon M.R., Reiner R.C., Sartorius B., Smith M., Topor-Madry R., Werdecker A., Vos T., Feigin V.L., Murray C.J.L. (2019). Global, regional, and national burden of multiple sclerosis 1990-2016: a systematic analysis for the global burden of disease study 2016. The Lancet. Neurology.

[bb0255] Montavon G., Samek W., Müller K.-R. (2018). Methods for interpreting and understanding deep neural networks. Digital Signal Process..

[bb0260] Mori S.S., Crain B.J. (2005). MRI Atlas of Human White Matter.

[bb0265] Nichols T.E., Borgwardt S.J., Kappos L., Kuster P., Mueller-Lenke N., Traud S., Smieskova R., Radue E.-W., Naegelin Y., Bendfeldt K., Klöppel S. (2012). Multivariate pattern classification of gray matter pathology in multiple sclerosis. NeuroImage.

[bb0270] Olah C., Mordvintsev A., Schubert L. (2017). Feature visualization. Distill.

[bb0275] Orrù G., Pettersson-Yeo W., Marquand A.F., Sartori G., Mechelli A. (2012). Using support vector machine to identify imaging biomarkers of neurological and psychiatric disease: a critical review. Neurosci. Biobehav. Rev..

[bb0280] Pawlitzki M., Neumann J., Kaufmann J., Heidel J., Stadler E., Sweeney-Reed C., Sailer M., Schreiber S. (2017). Loss of corticospinal tract integrity in early ms disease stages. Neurol. Neuroimmunol. Neuroinammat..

[bb0285] Payan A., Montana G. (2015). Predicting Alzheimer's disease: a neuroimaging study with 3D convolutional neural networks. CoRR abs/1502.

[bb0290] Polman C.H., Reingold S.C., Banwell B., Clanet M., Cohen J.A., Filippi M., Fujihara K., Havrdova E., Hutchinson M., Kappos L., Lublin F.D., Montalban X., O'Connor P., Sandberg-Wollheim M., Thompson A.J., Waubant E., Weinshenker B., Wolinsky J.S. (2011). Diagnostic criteria for multiple sclerosis: 2010 revisions to the McDonald criteria. Ann. Neurol..

[bb0295] Rajpurkar P., Irvin J., Bagul A., Ding D., Duan T., Mehta H., Yang B., Zhu K., Laird D., Ball R.L., Langlotz C., Shpanskaya K., Lungren M.P., Ng A.Y. (2017). MURA: Large Dataset for Abnormality Detection in Musculoskeletal Radiographs. http://arxiv.org/abs/1712.06957.

[bb0300] Rajpurkar P., Irvin J., Zhu K., Yang B., Mehta H., Duan T., Ding D., Bagul A., Langlotz C., Shpanskaya K., Lungren M.P., Ng A.Y. (2017). CheXNet: Radiologist-Level Pneumonia Detection on Chest X-Rays with Deep Learning. http://arxiv.org/abs/1711.05225.

[bb0305] Reich D.S., Lucchinetti C.F., Calabresi P.A. (2018). Multiple sclerosis. N. Engl. J. Med..

[bb0310] Renard D., Castelnovo G., Campello C., Bouly S., Le Floch A., Thouvenot E., Waconge A., Taieb G. (2014). An MRI review of acquired corpus callosum lesions. J. Neurol. Neurosurg. Psychiatry.

[bb0315] Rieke J., Eitel F., Weygandt M., Haynes J.-D., Ritter K. (2018). Visualizing convolutional networks for mri-based diagnosis of alzheimers disease. Understanding and Interpreting Machine Learning in Medical Image Computing Applications.

[bb0320] Roosendaal S.D., Geurts J.J.G., Vrenken H., Hulst H.E., Cover K.S., Castelijns J.A., Pouwels P.J.W., Barkhof F. (2009). Regional DTI differences in multiple sclerosis patients. NeuroImage.

[bb0325] Samek W., Binder A., Montavon G., Lapuschkin S., Mller K. (2017). Evaluating the visualization of what a deep neural network has learned. IEEE Transactions on Neural Networks and Learning Systems.

[bb0330] Samek W., Wiegand T., Müller K.-R. (2017). Explainable Artificial Intelligence: Understanding, Visualizing and Interpreting Deep Learning Models. https://arxiv.org/abs/1708.08296.

[bb0335] Schmidt P. (2017). Bayesian Inference for Structured Additive Regression Models for Large-Scale Problems with Applications to Medical Imaging.

[bb0340] Shrikumar A., Greenside P., Kundaje A. (2017). Learning important features through propagating activation differences. CoRR abs/1704.

[bb0345] Simonyan K., Zisserman A. (2014). Two-stream convolutional networks for action recognition in videos. Advances in Neural Information Processing Systems.

[bb0350] Simonyan K., Vedaldi A., Zisserman A. (2013). Deep inside Convolutional Networks: Visualising Image Classification Models and Saliency Maps. https://arxiv.org/abs/1312.6034.

[bb0355] Sinnecker T., Clarke M.A., Meier D., Enzinger C., Calabrese M., De Stefano N., Pitiot A., Giorgio A., Schoonheim M.M., Paul F., Pawlak M.A., Schmidt R., Kappos L., Montalban X., Rovira A., Evangelou N., Wuerfel J., for the MAGNIMS Study Group (2019). Evaluation of the central vein sign as a diagnostic imaging biomarker in multiple sclerosis. JAMA Neurol..

[bb0360] Solomon A.J., Watts R., Dewey B.E., Reich D.S. (2017). Mri evaluation of thalamic volume differentiates ms from common mimics. Neurol. Neuroimmunol. Neuroinammat..

[bb0365] Springenberg J.T., Dosovitskiy A., Brox T., Riedmiller M. (2015). Striving for simplicity: the all convolutional net. ICLR.

[bb0370] Suk H.-I., Lee S.-W., Shen D. (2014). Hierarchical feature representation and multimodal fusion with deep learning for AD/MCI diagnosis. NeuroImage.

[bb0375] Sundararajan M., Taly A., Yan Q. (2017). Axiomatic attribution for deep networks. CoRR abs/1703.

[bb0380] Tajbakhsh N., Shin J.Y., Gurudu S.R., Hurst R.T., Kendall C.B., Gotway M.B., Liang J. (2016). Convolutional neural networks for medical image analysis: full training or fine tuning?. IEEE Trans. Med. Imaging.

[bb0385] Thomas A.G., Koumellis P., Dineen R.A. (2011). The fornix in health and disease: an imaging review. RadioGraphics.

[bb0390] Thomas A.W., Heekeren H.R., Müller K.-R., Samek W. (2018). Interpretable LSTMs for Whole-Brain Neuroimaging Analyses. http://arxiv.org/abs/1810.09945.

[bb0395] Thompson A.J., Banwell B.L., Barkhof F., Carroll W.M., Coetzee T., Comi G., Correale J., Fazekas F., Filippi M., Freedman M.S., Fujihara K., Galetta S.L., Hartung H.P., Kappos L., Lublin F.D., Marrie R.A., Miller A.E., Miller D.H., Montalban X., Mowry E.M., Sorensen P.S., Tintoré M., Traboulsee A.L., Trojano M., Uitdehaag B.M.J., Vukusic S., Waubant E., Weinshenker B.G., Reingold S.C., Cohen J.A. (2018). Diagnosis of multiple sclerosis: 2017 revisions of the McDonald criteria. The Lancet. Neurology.

[bb0400] Tustison N.J., Avants B.B., Cook P.A., Zheng Y., Egan A., Yushkevich P.A., Gee J.C. (2010). N4itk: improved n3 bias correction. IEEE Trans. Med. Imaging.

[bb0405] Valverde S., Oliver A., Lladó X. (2014). A white matter lesion-filling approach to improve brain tissue volume measurements. NeuroImage.

[bb0410] Valverde S., Cabezas M., Roura E., González-Villà S., Pareto D., Vilanova J.C., Ramió -Torrentà L., Rovira À., Oliver A., Lladó X. (2017). Improving automated multiple sclerosis lesion segmentation with a cascaded 3D convolutional neural network approach. NeuroImage.

[bb0415] Vieira S., Pinaya W.H., Mechelli A. (2017). Using deep learning to investigate the neuroimaging correlates of psychiatric and neurological disorders: methods and applications. Neurosci. Biobehav. Rev..

[bb0420] Wang S.-H., Tang C., Sun J., Yang J., Huang C., Phillips P., Zhang Y.-D. (2018). Multiple sclerosis identification by 14-layer convolutional neural network with batch normalization, dropout, and stochastic pooling. Frontiers in neuro science.

[bb0425] Weygandt M., Hackmack K., Pfüller C., Bellmann-Strobl J., Paul F., Zipp F., Haynes J.D. (2011). MRI pattern recognition in multiple sclerosis normal-appearing brain areas. PLoS One.

[bb0430] Weygandt M., Hummel H.-M., Schregel K., Ritter K., Allefeld C., Dommes E., Huppke P., Haynes J., Wuerfel J., Gärtner J. (2015). MRI-based diagnostic biomarkers for early onset pediatric multiple sclerosis. NeuroImage.

[bb0435] Woo C.W., Chang L.J., Lindquist M.A., Wager T.D. (2017). Building better biomarkers: brain models in translational neuroimaging. Nat. Neurosci..

[bb0440] Wottschel V., Alexander D.C., Kwok P.P., Chard D.T., Stromillo M.L., Stefano N.De, Thompson A.J., Miller D.H., Ciccarelli O. (2015). Predicting outcome in clinically isolated syndrome using machine learning. NeuroImage.

[bb0445] Wyman B.T., Harvey D.J., Crawford K., Bernstein M.A., Carmichael O., Cole P.E., Crane P.K., DeCarli C., Fox N.C., Gunter J.L., Hill D., Killiany R.J., Pachai C., Schwarz A.J., Schuff N., Senjem M.L., Suhy J., Thompson P.M., Weiner M., Jack C.R. (2013). Alzheimer's disease neuroimaging initiative, standardization of analysis sets for reporting results from ADNI MRI data. Alzheimers Dement..

[bb0450] Yoo Y., Tang L.W., Brosch T., Li D.K.B., Metz L., Traboulsee A., Tam R. (2016). Deep learning of brain lesion patterns for predicting future disease activity in patients with early symptoms of multiple sclerosis deep learning of lesion patterns for early MS activity prediction. LNCS.

[bb0455] Yoo Y., Tang L.Y., Brosch T., Li D.K., Kolind S., Vavasour I., Rauscher A., MacKay A.L., Traboulsee A., Tam R.C. (2018). Deep learning of joint myelin and T1w MRI features in normal-appearing brain tissue to distinguish between multiple sclerosis patients and healthy controls. NeuroImage.

[bb0460] Yushkevich P.A., Piven J., Hazlett H.C., Smith R.G., Ho S., Gee J.C., Gerig G. (2006). User-guided 3d active contour segmentation of anatomical structures: significantly improved efficiency and reliability. Neuroimage.

[bb0465] Zeiler M., Fergus R., Fleet D., Pajdla T., Schiele B., Tuytelaars T. (2014). Visualizing and understanding convolutional networks. Computer Vision ECCV 2014, Volume 8689 of Lecture Notes in Computer Science.

[bb0470] Zhang C., Liu Y., Han X.-m., Gu J.-b., Bakshi R., Han Z., Tian H.-j., Cao X. (2017). Correlation between white matter damage and gray matter lesions in multiple sclerosis patients. Neural Regen. Res..

[bb0475] Zintgraf L.M., Cohen T.S., Adel T., Welling M. (2017). Visualizing Deep Neural Network Decisions: Prediction Difference Analysis.

[bb0480] Zurita M., Montalba C., Labbé T., Cruz J.P., Dalboni da Rocha J., Tejos C., Ciampi E., Cárcamo C., Sitaram R., Uribe S. (2018). Characterization of relapsing remitting multiple sclerosis patients using support vector machine classifications of functional and diffusion MRI data. NeuroImage.

