# Supplementary material for: Uncovering convolutional neural network decisions for diagnosing multiple sclerosis on conventional MRI using layer-wise relevance propagation
Source: Neuroimage Clin. 2019 Sep 6;24:102003. doi: 10.1016/j.nicl.2019.102003 (PMC6807560; doi:10.1016/j.nicl.2019.102003)
Supplement: Supplementary file 1 — Supplementary material [file mmc1.pdf]

1. Supplementary Materials

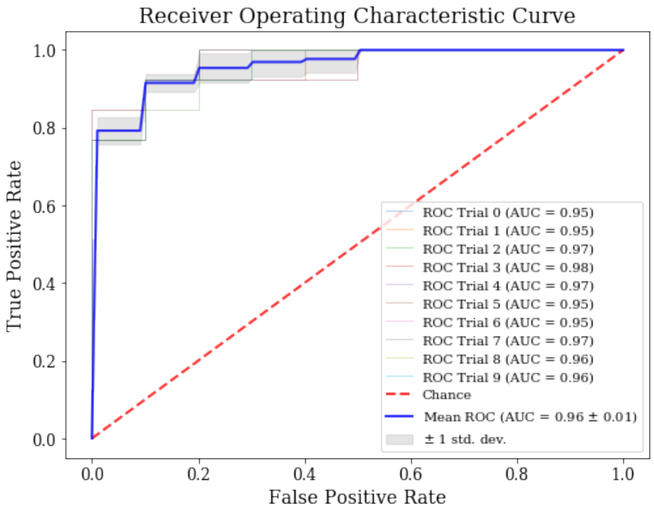

Figure 1: Performance of the pre-trained CNN model (fine-tuned on MS) on the holdout set.

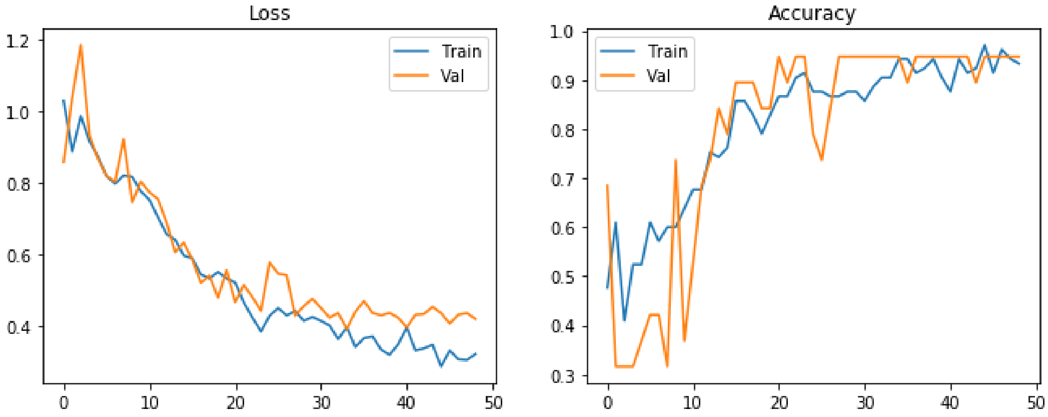

Figure 2: Training curve of fine-tuning the model on the MS data set. It shows the run of the model with the best validation score.

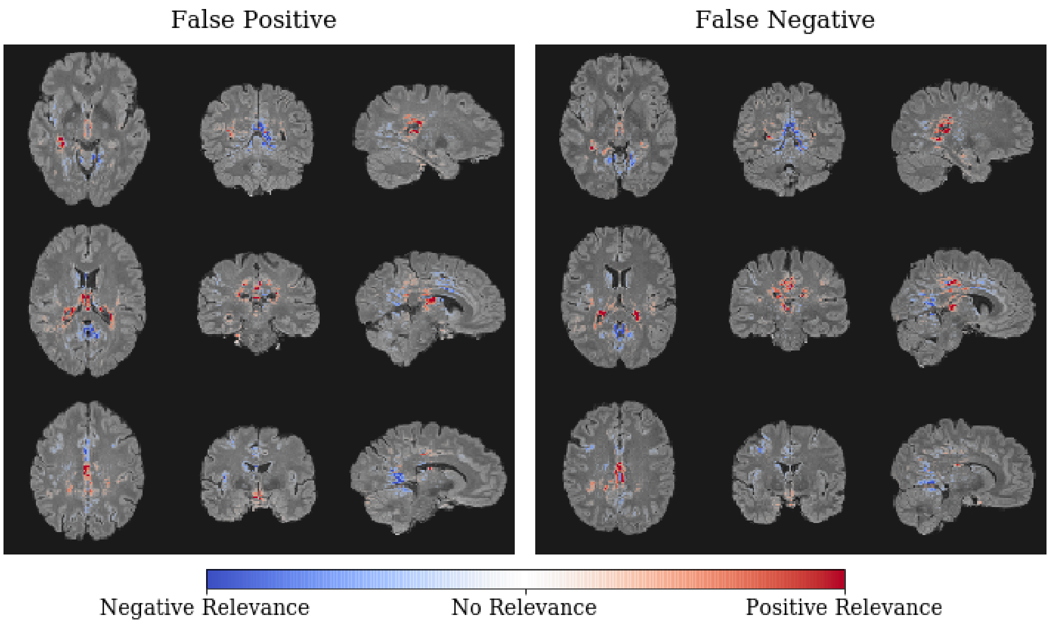

Figure 3: Individual LRP heatmaps (overlayed on the input FLAIR data) of the two misclassified subjects from the holdout set. Heatmap values are normalized in the range [-0.03, 0.03]. Colors indicate regions supporting (red) or rejecting (blue) the classification as a MS patient with respect to the underlying CNN model. For the false positive (healthy control classified as MS patient), it can be seen that the model focuses on hyperintensities in posterior ventricular regions (so-called dirty appearing white matter). For the false negative (MS patient classified as healthy control), we see that the heatmaps look rather similar to the heatmaps of correctly classified controls. Both subjects have only a low quantity of small lesions, which makes especially the MS patient hard to classify. The model has learned to distinguish subjects with higher quantities of lesions based on the location of the lesions (posterior periventricular vs. anterior periventricular and non-periventricular white matter lesions). Also, both scans have a normal score for being MS or healthy control, the false positive with a sigmoid value of 0.65 (holdout mean of true positives: 0.86) and the false negative with a sigmoid value of 0.36 (holdout mean of true negatives: 0.30). In order to identify specific deficiencies of the model a larger data set is required.

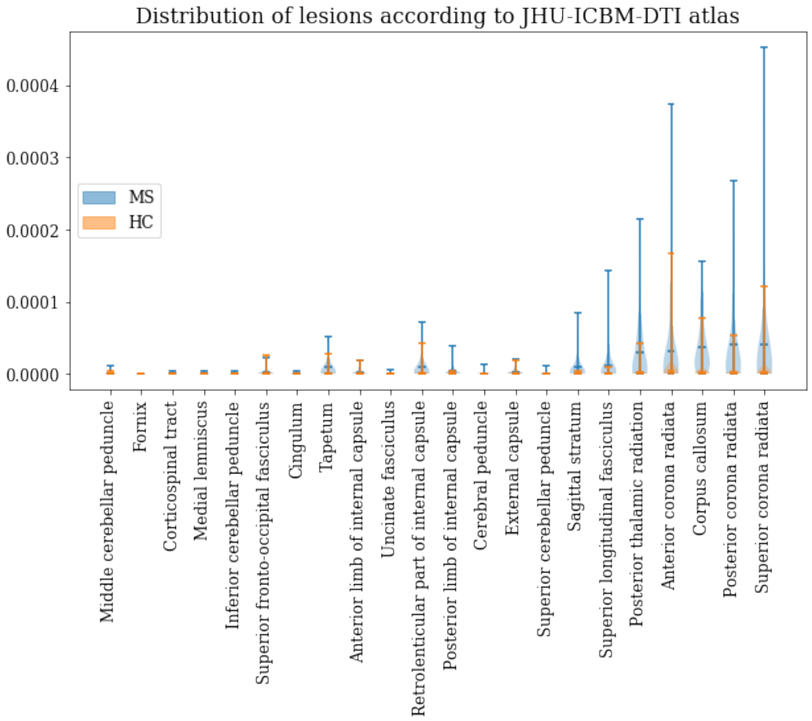

Figure 4: Lesion distribution over white matter areas from the JHU ICBM-DTI atlas, separately for MS patients and healthy controls (HC).

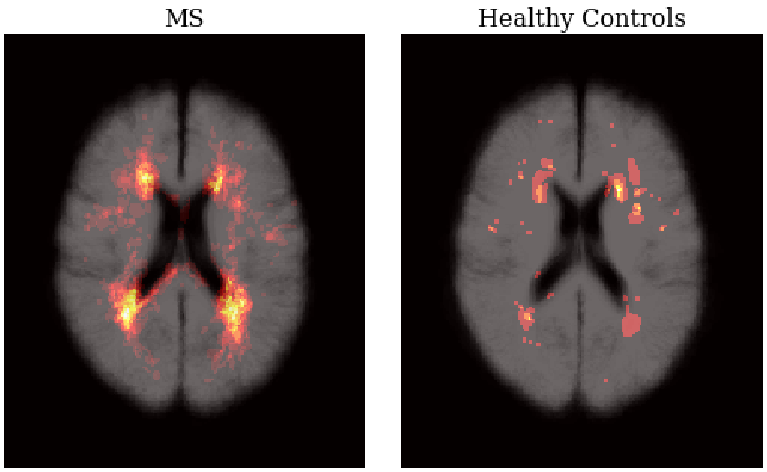

Figure 5: Lesion distribution in MS patients (left) and healthy controls (right).

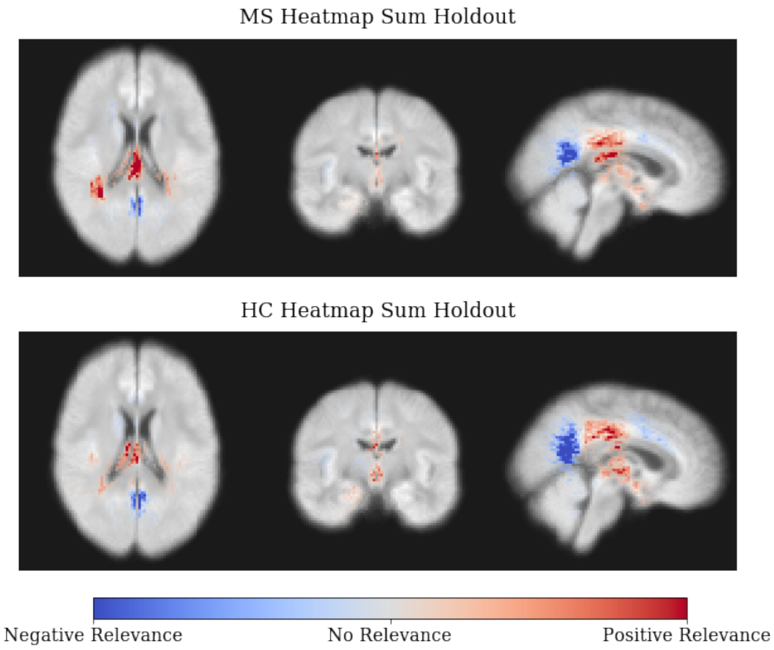

Figure 6: Average gradient*input heatmaps for all correctly classified MS patients (top) and all correctly classified healthy controls (bottom) in the holdout set. Values are normalized in the range [-0.02, 0.02]. Note the similarity with the LRP average in Figure 3

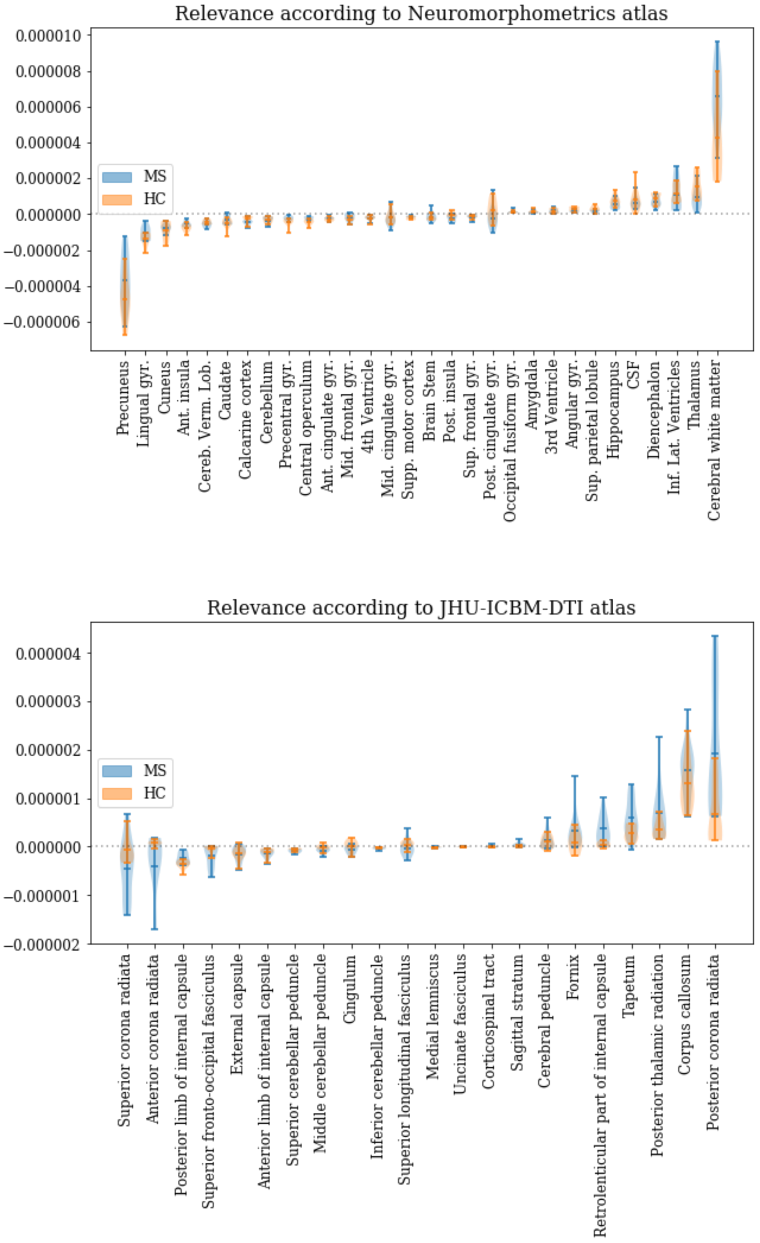

Figure 7: Gradient*input relevance distribution over (a) 30 (mainly) gray matter areas from the Neuromorphometrics atlas and (b) 22 white matter areas from the JHU ICBM-DTI atlas, separately for MS patients and healthy controls in the holdout set. Please note the similarity to the LRP distribution in Figure 5
